# MAR-mediated integration of plasmid vectors for *in vivo* gene transfer and regulation

**DOI:** 10.1186/1471-2199-14-26

**Published:** 2013-12-02

**Authors:** Stefania Puttini, Ruthger W van Zwieten, Damien Saugy, Małgorzata Lekka, Florence Hogger, Deborah Ley, Andrzej J Kulik, Nicolas Mermod

**Affiliations:** 1 Institute of Biotechnology, University of Lausanne, Lausanne, Switzerland; 2 The Henryk Niewodniczański Institute of Nuclear Physics, Polish Academy of Sciences, Kraków, Poland; 3 Laboratory of Physics of Living Matter - IPSB, Ecole Polytechnique Fédérale de Lausanne, Lausanne, Switzerland; 4 Laboratory for Molecular Biotechnology, Station 6, EPFL, 1015 Lausanne, Switzerland

**Keywords:** Inducible expression, Non-viral vectors, MAR elements, Erythropoietin, Utrophin, Gene therapy

## Abstract

**Background:**

The *in vivo* transfer of naked plasmid DNA into organs such as muscles is commonly used to assess the expression of prophylactic or therapeutic genes in animal disease models.

**Results:**

In this study, we devised vectors allowing a tight regulation of transgene expression in mice from such non-viral vectors using a doxycycline-controlled network of activator and repressor proteins. Using these vectors, we demonstrate proper physiological response as consequence of the induced expression of two therapeutically relevant proteins, namely erythropoietin and utrophin. Kinetic studies showed that the induction of transgene expression was only transient, unless epigenetic regulatory elements termed Matrix Attachment Regions, or MAR, were inserted upstream of the regulated promoters. Using episomal plasmid rescue and quantitative PCR assays, we observed that similar amounts of plasmids remained in muscles after electrotransfer with or without MAR elements, but that a significant portion had integrated into the muscle fiber chromosomes. Interestingly, the MAR elements were found to promote plasmid genomic integration but to oppose silencing effects *in vivo*, thereby mediating long-term expression.

**Conclusions:**

This study thus elucidates some of the determinants of transient or sustained expression from the use of non-viral regulated vectors in vivo.

## Background

Progress has been made on therapeutic gene regulation systems applicable to gene therapies, in which transgene expression is controlled pharmacologically by administering a small molecule drug. One of the favorite systems is based on the bacterial tetracycline-repressed TetR protein of *E. coli *[1,2]. It relies on inducer compounds with a proven history of medical safety, i.e. tetracycline and derivatives, which regulate the ability of engineered TetR protein derivatives to bind particular DNA sequences inserted in target promoters. With this system, regulation of the mouse erythropoietin gene has been achieved in mice muscles over periods of several months using non-viral or viral vectors. For instance, beta-thalassemic mice were rendered steadily normocythemic by intramuscular injection of a tetracycline-inducible AAV vector encoding mouse erythropoietin (EPO), and doxycycline dosage was used to control hematocrit [2]. No adverse immune response against the bacterial protein was noted. However “leaky” expression in absence of the tetracycline derivative was often noted, especially at high transgene copy numbers [3,4].

To alleviate this problem, several improved versions have been developed, whereby activators and repressors of gene expression are combined [5-8]. These new versions display little or no basal activity, while they maintain induced expression at levels sufficient to achieve physiological effects in rodents. One of the tightest genetic switch systems for controlled transgene transcription relies on chimerical repressor and activator proteins functioning in a regulatory network [6,9]. The hallmark of this system is the efficient transgene silencing in the OFF-state, while addition of the inducer drug allowed for robust activation of transgene expression *in vitro* and *in vivo *[10].

Regulated EPO expression and hematocrit control were achieved for periods up to one year in normal C57Bl6 mice, without detectable immune response [11], although induced expression has occasionally been found to decline with time *in vivo*. Gene regulation was also achieved with these vectors after DNA transfer in non-human primates, but with a reduced regulatory window as compared to mice. Another limitation observed in long-term experiments has been the potential immunogenicity of some of the Tet-derived proteins in monkeys, which can also hinder long term expression regulation [11-13].

In addition to improvements of the DNA vector elements, gene delivery strategies have also been under intense scrutiny over the past years with regard to therapeutic gene transfer, with viral vectors being most prominent. For instance, retroviral or lentiviral vectors have been used widely as they mediate efficient transduction into the cell nucleus and transgene integration into the cell genome. However, such viral vectors often integrate preferentially into or near cellular genes, which has caused adverse effects in clinical trials [14]. As of yet, such tropism has not been reported for non-viral vectors. Thus, non-viral gene transfer is also currently considered as an alternative, especially with non-integrating vectors or with vectors that do not integrate preferentially into genes [15].

Recombinant AAV vectors can efficiently transduce muscles after systemic administration in normal mice [16], and AAV vectors carrying a microdystrophin gene were shown to correct the phenotype of dystrophic *mdx* mice [17]. However, the AAV capsid cannot accommodate a cargo size like full-length utrophin or dystrophin cDNA, and novel AAV vectors were thus developed to increase the transgene capacity by *trans*-splicing [18]. This system takes advantage of AAV’s ability to form head-to-tail concatemers by the recombination of the ITRs. In this approach, the transgene cassette is split between two rAAV vectors containing adequately placed splice donor and acceptor sites. Transcription from two rAAV vectors followed by correct *trans*-splicing of the mRNA transcripts can results in a functional gene product. This application can be used to deliver therapeutic genes up to 9 kb in size, which is not sufficient to confer full-length dystrophin or utrophin expression.

Non-viral vectors like plasmids can accommodate coding sequences such as that of full-length dystrophin, as can be required to fully correct human dystrophy, but they are subject to a lower efficiency of DNA transfer. One of the most efficient physical DNA transfer methods *in vivo* consists of the electrotransfer approach, which is also referred to as *in vivo* electroporation [19]. DNA is injected into an organ or tissue of choice, and an electrical field is applied to promote or increase the entry of the DNA into the cells. Although systemic intramuscular treatment is not feasible, localized electroporation has often been used to achieve transient or more persistent expression of genes of interest in animal disease models, and it has also been used in humans, in clinical trials [19,20].

The purpose of this study was to achieve and document tight transgene expression regulation in an animal model of muscle diseases using non-viral vectors. We report that sustained transgene regulation requires matrix attachment regions (MAR) epigenetic regulatory sequences, and we show that these elements not only favor stable and long-term expression but that they also promote the chromosomal integration of the transgenes *in vivo*.

## Results

### Establishment of optimal conditions for *in vivo* transgene control in mice muscles

**Figure 1:**
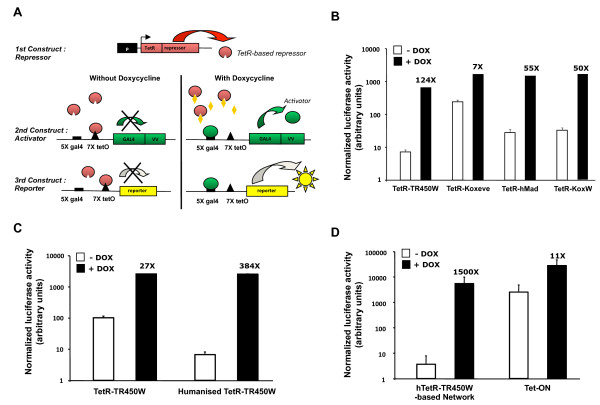
**Construction of a gene network for regulated transgene expression *in vivo. *(A)** The regulatory network-based expression system assessed in this study consists of 3 plasmid components encoding a constitutively expressed repressor, an inducible activator, and a reporter or therapeutic gene regulated by both the repressor and the activator proteins. Without doxycycline, the repressor protein binds to and represses the promoters of the two other plasmids. Doxycycline binds to and inactivates the repressor, which relieves the activator promoter from repression and allows low expression levels to establish. As the activator is expressed, it activates it own expression as well as that of the reporter gene, gradually increasing expression to maximally induced levels. **(B)** The strength of various repressor constructs was evaluated by the electrotransfer of the network’s three plasmid components into the tibialis anterior muscle of wild type mice, using the luciferase coding sequence as a reporter gene. Mice were provided doxycycline (+ dox) or not (- dox) in the drinking water and they were sacrificed after two weeks. The relative luciferase activity was determined from muscle extracts. Numbers indicate the fold-induction of normalized luciferase activities by doxycycline. **(C)** The strength of the prokaryotic part of the chimeric TetR-TR450W repressor was compared when expressing the fusion protein either from the bacterial DNA sequence of from a DNA sequence with “humanized” codons [24]. **(D)** Performance of the network system controlled by the humanized TetR (hTetR) fused to the TR450W repressor domain expressed from the β actin promoter in mice muscles, as a comparison to that obtained from the original pTet-ON™ system (Clontech) electrotransferred similarly. Fold activations obtained upon the addition of doxycyclin are as indicated above each filled bar. Each column represents the mean of values obtained from 16 muscles of 8 mice per condition.

The regulatory network system used in this study consists of a constitutively expressed TetR-based transcriptional repressor that inhibits the expression of both an activator gene and of the gene of interest in the absence of doxycycline. Addition of doxycycline inactivates the repressor, relieving the expression of the transcriptional activator, which will in turn activate its own expression in a positive feed-back loop, and as well as that of a therapeutic or reporter gene (Figure 1A). Previous work has shown that this system can yield tight transgene expression regulation in stable cell lines in culture [6,9]. The efficiency of this regulatory network *in vivo* was quantified first using a reporter gene encoding luciferase. Plasmids encoding the 3 components of the network (i.e. the repressor, activator and reporter) were electrotransferred in the tibial cranial muscles of C57Bl/6 mice, and luciferase activity was assayed from muscle extracts. Initial assays using the network components, as previously characterized and optimized in cultured cells, yielded a relatively smaller regulatory window *in vivo* (data not shown). Thus, we set up to improve the performance of this network for in vivo use, as described below.

One of the improvements involved the identification of a more potent repressor protein, coupled to a constitutive promoter that was not subjected to silencing effects *in vivo* and that had a suitable strength. Various repressor constructs were generated from previously characterized potent transcriptional repression domains, such as the hormone-insensitive truncated version of the hormone-binding domain of the thyroid hormone receptor fused to the hairy protein tetrapeptide WRPW (TR450W, [9]). Alternatively, we used the KRAB repressor domains of Kox1 [21] of Even-skipped (koxeve) [22], or a fusion of the WRPW tetrapeptide and of the SID domain of human hMad (koxW) [23]. Expression of these repressors from the cellular β actin promoter and co-transfer with the 2 other components of the network yielded comparable expression levels upon the addition of doxycycline to the drinking water of mice (Figure 1B). However, without doxycycline, the TR450W repressor provided a more tightly repressed expression in the OFF state.

We also compared the strength of the KoxW repressor domain fused either to the original bacterial TetR sequence or to a version engineered to contain a humanized coding sequence, for more efficient expression in mammalian organisms (hTetR) [24]. As expected, there was no difference when comparing the transgene expression levels as induced by the presence of doxycycline, when the two repressors are in an inactive state. However, in the absence of doxycyclin, the humanized TetR version conferred a 10-fold tighter repression of the regulatory system (Figure 1C). When expressed from the chicken β-actin promoter, the fusion of the humanized hTETR moiety to the TR450W repressor domain provided a dose dependent repression of expression that proved superior to that mediated by alternative repressors (Additional file 1 and data not shown). When combining all these improvements, the regulatory network provided high induced expression without compromising tightness, yielding a 1’500-fold regulatory window in muscles *in vivo* (Figure 1D).

### Regulated expression of a therapeutic transgene in mouse muscles

We next assessed whether the regulatory network may mediate expression at levels sufficiently high to mediate a functional response from diseased muscle fibers. Utrophin is a protein homologous to dystrophin, the protein missing in Duchenne muscular dystrophy (DMD) patients. High-level expression of the fetal utrophin protein was shown to substitute dystrophin and to yield sustained muscle function without a potential immune response in the *mdx* mouse model of DMD that lacks dystrophin [25]. The large size of the utrophin or dystrophin coding sequences exceeds the capacity of viral vectors, and although certain high capacity adenoviral vectors could circumvent this limitation, these vectors are still hampered by immunogenicity and gene transfer problems in adult muscles [26]. Therefore, current clinically relevant viral vectors are only able to accommodate truncated forms of these therapeutic proteins, with the perspective to convert Duchenne muscular dystrophy into relatively milder Becker dystrophies [27].

**Figure 2:**
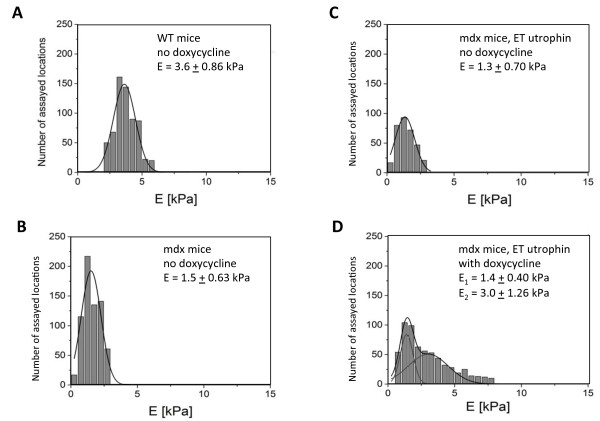
**Regulated utrophin expression in *in vivo *electroporated mouse muscles.** Mouse muscles were electroporated with the optimized version of Network system, consisting of the pCAG-htetR-TR450W repressor, 5Xgal4-7XtetO-Gal4VV activator and 5Xgal4-7XtetO-mUtrophin reporter constructs. The therapeutic efficacy of regulated utrophin expression in the muscles of *mdx* mice was assessed by atomic force microscopy [AFM, as described by Puttini et al. (2009)]. AFM assays of muscles from wild type (WT) or dystrophic (*mdx*) mice were performed as controls **(A, B)**. Alternatively, 8 weeks *mdx* mouse muscles were assayed two weeks after the electrotransfer of the regulatory network containing the utrophin cDNA as a therapeutic transgene, and with the addition or not of doxycycline in the mice drinking water, as indicated **(C, D)**. Muscle explant sections were immobilized and thereafter assayed using the AFM pressing mode to determine the force required to reach a given indentation in individual muscle fibers, as an assay of the expression of sufficient levels of functional utrophin to restore a normal resistance to the muscle fibers sarcolemmal membrane [29]. Results of several hundreds of independent measurements performed on distinct section locations and myofibers are represented as the distribution of Young’s modulus values in histograms constructed with a bin size of 0.5 kPa, which quantitatively describe the muscle’s resistance to mechanical deformation. Here, we pooled the results from 4 independent experiments, each one consisting of 2 electroporated animals, one treated with dox and the other one left untreated. Each animal had an 1 electrotransfer in each of the two tibialis muscles, and each muscle was processed into at least 2 sections, so a minimum of 4 muscle sections were measured per condition and experiment.

However, non-viral plasmid DNA vectors are not subjected to similar limitations, and this approach was shown to yield up to 50% of the muscle fibers expressing a GFP transgene, which should be sufficient to achieve a therapeutically effective treatment of skeletal muscles [28,29]. Thus, expression of utrophin from the regulatory network was evaluated after *in vivo* electrotransfer of the tibialis anterior muscles of dystrophic *mdx* mice. As previously reported, utrophin cDNA transfer can elevate utrophin protein levels to about 2-fold with the current electroporation protocol [29]. Here, the therapeutic efficacy of utrophin expression from the regulatory network was evaluated by Atomic Force Microscopy (AFM), as it was previously shown to provide a sensitive approach to reveal the recovery of normal muscle resistance and strength upon utrophin or dystrophin expression from the assay of single muscle fibers [29]. Fifteen days after electrotransfer and doxycycline induction, some of the *mdx* muscle fibers were found to be more resistant to elastic deformation than untreated *mdx* fibers, yielding Young’s Modulus elasticity values in the 2 to 6 kPa range typical of healthy murine muscles (Figure 2A and D). Modeling of the distribution of the Young’s Modulus values of individual muscle fibers indicated that approximately 50% of the fibers had increased mechanical resistance, with an average of 2.5 kPa. This is similar to what was previously observed when expressing utrophin from the strong cytomegalovirus (CMV) promoter [29]. We concluded that the inducible system allowed the functional recovery of the muscle fiber sarcolemmal membrane, and that the network expression system may therefore confer sufficient utrophin expression to mediate a therapeutic effect.

### MAR elements mediate sustained transgene expression in muscle *in vivo*

Epigenetic regulatory DNA sequences termed MAR were previously linked to a wide diversity of processes, including the attachment of chromatin to the nuclear matrix, chromatin remodeling, and stabilization of transgene expression [10,30]. They have been shown to mediate expression from episomal replicating vectors when transcribed, however they were also associated to an increased genomic integration of transgenes when incorporated into plasmid expression vectors upstream of the promoter [31,32]. We previously identified two potent human MAR (hMAR) elements from chromosome 1 and X, termed MAR 1–68 and X-29 respectively, that each could sustain GFP expression in cultured cells, including muscle stem cells, and in mouse muscles after transplantation and differentiation [9], Van Zwieten et al., submitted for publication]. Also, these MARs mediated elevated levels of circulating red blood cells upon the expression of a secreted protein, erythropoietin (EPO) [10]. However, the mode of action of these MARs and the duration and levels of transgene expression after *in vivo* electrotransfer have not been characterized.

**Figure 3:**
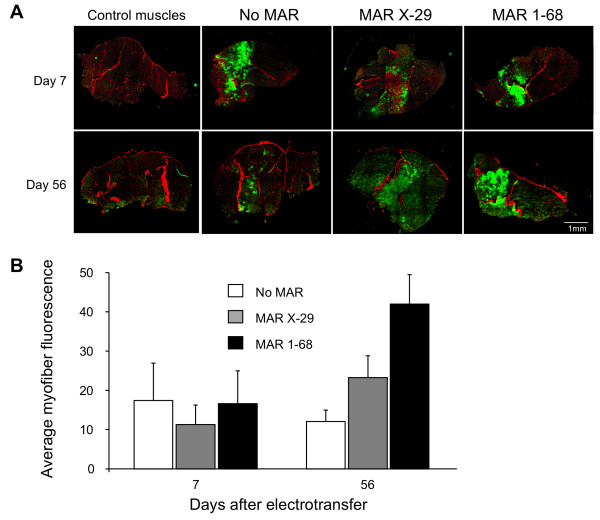
**Expression of MAR-driven GFP genes after mouse muscle electrotransfer.** Wild-type mice muscles were injected with 30 μg of GFP expression plasmids containing or not a MAR element, or injected with saline for control muscles, as indicated, prior to electrotransfer. Muscles were collected 7 or 56 days after the electrotransfer. Representative transversal muscle sections illustrate the background and GFP fluorescence in green and the extracellular matrix proteins with Wheat Germ Agglutinin separating individual myofibers in red colors **(A)**. The GFP fluorescence of single myofibers was quantified in 4 muscle sections from 2 mice per condition, and they are represented as the average and standard deviation of the GFP fluorescence of individual myofibers, after the subtraction of the background fluorescence values determined from the control muscles **(B)**.

To assay the effect of MAR 1–68 and X-29 in mouse muscles, constitutive GFP expression vectors containing or not the MAR upstream of the promoter were electrotransferred into the muscles of C57Bl/6 mice as before. This revealed an average of approximately 50% of GFP-positive fibers 7 days after the electrotransfer, irrespective of the inclusion or not of a MAR (Figure 3A). However at day 56 post-electrotransfer, only muscles injected with the vectors containing the human MAR (hMAR) 1–68 and hMAR X-29 showed sustained and higher GFP expression levels. When the average GFP fluorescence levels were quantified in the myofibers of whole muscle sections, we found that the expression levels had even increased with hMAR 1–68, which is reminiscent of the ability of this MAR to reverse transgene silencing in cultured cell lines [33]. Two other MAR elements of human or murine origin evaluated in parallel did not confer similarly elevated and fully sustained long-term expression (data not shown). The hMAR 1–68 was therefore selected for inclusion into the final versions of the network plasmids, upstream of the regulated promoters that drive the expression of the activator and therapeutic genes in the 5XGal4-7XtetO-Gal4VV and 5XGal4-7XtetO constructs, respectively, in the following experiments.

### hMAR 1–68 mediates persistent expression

**Figure 4:**
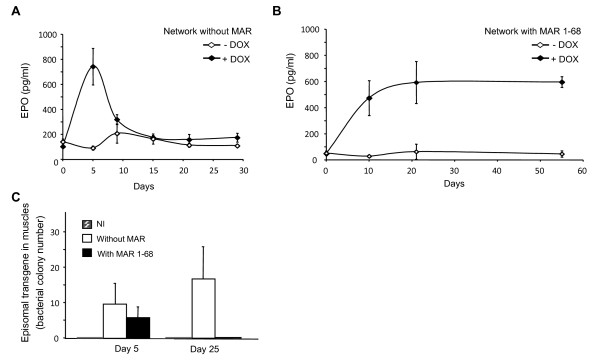
**Sustained expression of MAR-containing expression vectors. (A, B)** Mice muscles were electrotransferred with EPO-expressing network plasmids containing or not hMAR 1–68, and the blood plasma of mice treated with doxycycline or not was used to quantify EPO levels by ELISA. **(C)** Mice similarly treated as those of panels **A** and **B** were sacrificed 5 or 25 days post-electrotransfer, and total DNA was extracted from the electrotransferred muscles. Episomal plasmid rescue was performed by transforming competent bacteria with a fraction of the total DNA extract and by scoring the occurrence of ampicillin-resistant colonies on Petri dishes.

We next assessed the kinetics of transgene expression by probing circulating levels of the secreted murine EPO protein when expressed from the regulatory network. Maximal induction of EPO levels in the blood plasma of electrotransferred mice was obtained after 5 days of doxycycline treatment in the absence of the MAR. Thereafter, expression decreased rapidly, with EPO levels dropping to levels such as those of non-induced mice after 15 days (Figure 4A, and data not shown). When the hMAR1-68 was placed in front of the inducible promoters driving the expression of the activator and of the EPO gene, sustained expression was obtained in the presence of doxycycline, while expression levels in the off-state were not perturbed by inclusion of the hMAR (Figure 4B).

As expression levels from the constitutive CAG promoter driven repressor construct were sufficient for sustained inhibition of the regulated promoters in the OFF-state *in vivo*, inclusion of a MAR in this plasmid vector was deemed unnecessary for the efficiency of the hTetR-TR450W-based network system. This is consistent with our previous observations that constitutive expression from another strong promoter was not prone to loss-of-expression effects in electrotransferred mice muscles [10].

We hypothesized that the long-term expression observed from the regulated promoters in the presence of the MAR might either result from a protection against silencing effects, or from the abrogation of plasmid loss. This was assessed by quantifying the number of episomal plasmid copies remaining in the mouse muscles after the electrotransfer of GFP-expression plasmids. Total DNA was extracted from electrotransferred mouse muscles, and a “plasmid rescue” experiment was performed. This consisted of transforming electro-competent bacterial cells with aliquots of the total DNA extracts, followed by quantification of ampicillin-resistant colonies after plating. One day after electrotransfer of the MAR-devoid plasmids, the number of ampicillin-resistant colonies was 30-fold higher than after 5 days, irrespective of the inclusion of a MAR element (Figure 4C and data not shown). We therefore concluded that most injected plasmids were quickly lost, as expected from the previously reported fast lymphatic draining of plasmids remaining in the interstitial space surrounding muscle fibers [19]. Thereafter, the number of colonies obtained from MAR-devoid constructs remained stable for over 3 months, indicating that the remaining plasmids were stably maintained as episomal structures in the muscle.

Surprisingly, when the hMAR 1-68-containing plasmids were tested in the plasmid rescue assay, episomal structures were quickly lost, being lower 5 days post-electrotransfer and becoming undetectable after 25 days (Figure 4C), despite the persistent muscular transgene expression levels. When plasmids were mixed to total genomic DNA extracted from non-electrotransferred muscles followed by bacterial transformation, similar bacterial colony counts were obtained with plasmids containing or not the hMAR (Additional file 2). This indicated that the mere presence of the MAR or plasmid size differences could not explain the total loss of detectable episomal MAR-containing plasmids 25 days after *in vivo* electroporation. As the expression plasmid DNA must still be present, since stable and high EPO levels are still being expressed (Figure 4B), we reasoned that the MAR-containing plasmids might become entangled in some cellular structure or may form large concatemers, such that they would not be recovered in muscle extracts and/or not transform in bacteria because of their large size. Alternatively, the hMAR might have promoted plasmid integration into the genome of muscle fibers, converting the episomal transgenes into chromosomally integrated structures.

### Persistence of hMAR 1-68-containing plasmids in murine muscles

**Figure 5:**
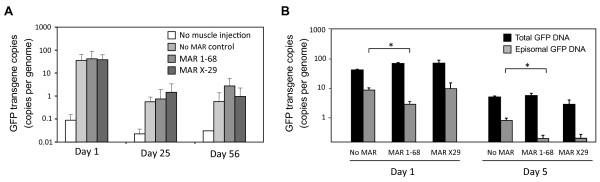
**Total and episomal transgene DNA quantification. (A)** Real-time PCR quantification of total GFP transgenes after muscle electrotransfer. GFP expression vector containing the indicated MAR element were introduced in mice muscles by electrotransfer. At the indicated day after the electrotransfer, total DNA extracts were prepared from the muscles. 120 ng of total DNA was used for qPCR assays. GFP copy numbers were normalised to those of the cellular GAPDH gene, and they are represented as the average number of GFP gene copies per diploid myofiber nuclei. **(B)** Muscles of mice were collected 1 or 5 day after the electrotransfer of GFP-expression plasmids containing or not a MAR element, as indicated. Total DNA was extracted and processed to quantify the GFP transgene and GAPDH cellular gene by quantitative PCR (total GFP DNA). Alternatively, the DNA preparations were treated with the PS-DNase exonuclease to degrade all transgenes occurring as chromosomally integrated linear DNA molecules (Episomal GFP DNA). GFP DNA amounts were normalized to those of GAPDH prior to PS-DNase treatment, and they are expressed as the average number of copies per diploid myofiber nucleus, after substraction of the background non-specific values obtained for not injected muscles.

MAR elements have been implicated with an activation of homologous recombination and with the formation of plasmid concatemers in transfected cell lines [31]. Persistence of the electrotransferred DNA was assessed by evaluating the total number of copies of GFP transgene in mouse muscle extracts by quantitative PCR (qPCR), as well as the fraction of it remaining in circular structures. The total transgene copy number was comparable throughout a time course, when comparing constructs containing or not a MAR after muscle electrotransfer (Figure 5A). When the GFP transgene copy number was normalized to that of the chromosomal GAPDH gene, high transgene copies per diploid genome were found at day 1, followed by a stable but much lower occurrence of transgene copies per genome thereafter, as found earlier with the plasmid rescue assay (Figure 5A and data not shown). We concluded from these findings that the loss of recoverable MAR-containing plasmids cannot be attributed to a preferential loss of the vector DNA or from the disappearance of MAR-containing muscle fibers, but that it may relate to the episomal status of the various vectors.

Thus, we next assessed whether the disappearance of recoverable episomal plasmids in presence of the MAR might result from the chromosomal integration of the vectors. To do so, we treated all DNA extract samples with PS-DNase (Plasmid-safe DNase), a strict exonuclease that allows the digestion of all linear chromosomal DNA, but that does not digest episomal circular DNA structures, as demonstrated for instance for adenovirus-associated viral genomes and derived vectors extracted from mouse, primate and human muscles [34-36]. Selectivity of the nuclease was ascertained in control studies performed on total genomic DNA spiked with circular or linear plasmids, as well as on chromosomally-integrated GFP expression vectors. This indicated that 99% or more of the linear DNAs were digested by the PS-DNase, while more than 90% of the circular plasmids were not (Additional file 3).

One day after electrotransfer, digestion with the PS-DNase indicated the occurrence of an average of approximately 10 copies per genome of circular plasmids when devoid of a MAR, while this was decreased to 2 copies in presence of hMAR 1–68 (Figure 5B). As a similar number of total GFP gene copies was obtained when the chromosomal DNA was not digested with the PS-DNase, we concluded that the hMAR 1-68-containing plasmid had integrated into cellular chromosomes with a higher frequency. 5 days after the electrotransfer, the amount of MAR-containing episomal vectors dropped to levels close to the detection limits, implying that most if not all plasmids had integrated in the genome (Figure 5B and Additional file 3). Although some of the MAR-devoid plasmids remained in episomal form, a proportion of it appeared to have integrated chromosomally as well. Interestingly, hMAR X-29 appeared to mediate a slower rate of plasmid integration as compared to hMAR 1–68, as it was indistinguishable from the MAR-devoid plasmid at day 1, while it was essentially completely integrated at day 5.

Overall, this indicated that MAR-devoid plasmids can persist as episomes after muscle electrotransfer, while MAR-containing constructs remain at comparable copy number, but in a form that may be chromosomally integrated. Interestingly, both types of vectors were retained at a comparable number of copies in the long term, stabilizing around 1–10 copies per genome on average (Figure 5A), but expression persisted only for the MAR-containing plasmids.

## Discussion

In this study, we wished to optimize and document sets of vectors for regulated *in vivo* expression that rely on an activator and a repressor protein functioning in a network regulated by doxycycline, a tetracycline antibiotic derivative commonly used to treat infections in humans. We found that a wide regulatory window can be obtained from this network once constituents of the regulatory system were adapted and optimized for *in vivo* use. Notably, we used a humanized TetR, optimized the repressor strength and used a constitutive cellular promoter that showed no detectable epigenetic silencing. We also demonstrated that this regulated gene expression system can be used to express therapeutic transgenes *in vivo* at levels reaching physiological relevance for the utrophin myofiber protein and for the murine EPO circulating hormone.

We also found that induced expression was quite short-lived in the regulated hTetR-TR450W network when circulating EPO levels were quantified from blood drawn from the same mice at distinct times after electrotransfer. This loss-of-expression effect could be counteracted by the inclusion of a MAR element next to the regulated promoters, allowing persistent EPO expression in inducing conditions, and yet without altering the tight repression observed without doxycycline. We also found that inclusion of MAR elements in the plasmids not only allowed persistent expression, but that this was accompanied by the rapid loss of episomal vector and by the likely chromosomal integration of the electrotransferred plamids. Similar observations were made using vectors mediating either regulated or constitutive expression, when expressing various types of recombinant proteins of either heterologous (GFP) or autologous (EPO) origin, when using several distinct MAR elements, and when assessing the fate of the transferred plasmids by unrelated assays (the rescue of episomal plasmids or the degradation of the chromosomal DNA).

Early studies exploring the fate of plasmids after muscle electrotransfer obtained no evidence of chromosomal integration of plasmids [37]. However, later and more sensitive assays indicated that plasmids do integrate upon electrotransfer, although a significant proportion of the plasmid vector can remain extra-chromosomal [38]. Here we find that chromosomal integration may be significantly increased upon addition of MAR elements in plasmid vectors, and that only trace amounts of the plasmids may remain in circular episomal form when containing the hMAR 1–68. These results are reminiscent of recent observations that hMAR 1–68 can promote and significantly increase the integration of plasmids in the chromosomes of cultured CHO cells during stable transfections [31]. Here, we find that this MAR may have a similar integration-promoting activity in muscle fibers *in vivo*, and that it may be more potent in this respect when compared to other human MARs such as hMAR X-29. Grandjean et al. (2011) showed that MARs promote plasmid integration by a homologous recombination-related mechanism, while Wang et al. (2004) showed that electrotransferred plasmids often integrate at AT-rich chromosomal loci. As the MAR elements such as those used in this study are prominently AT-rich, it will be interesting to determine if plasmid-borne MARs may promote plasmid integration at genomic loci containing homologous cellular MAR elements. Whether plasmids containing a MAR, as electrotransferred in our assays, have a tropism for intragenic regions remains unknown, as our sequencing efforts directed at identifying transgene integration sites with a MAR were unsuccessful. Thus, a potential genotoxicity of genome-integrated MAR-containing plasmids cannot be excluded at present. Nevertheless, the hTetR-TR450W based network inducible system was evaluated at several time points and up to 9 months, during which no adverse effects on the health nor tumorigenic effect was noticed in all tested conditions for over 100 mice altogether.

In this study, we have devised a novel method to assess more quantitatively the fate of plasmids upon electrotransfer, as based on the use of a strict exonuclease coupled to real time quantitative PCR assays. Results obtained with this approach suggested that plasmid integration may be more frequent than previously considered. For instance, Wang et al. (2004) reported that integration events remain infrequent, and that this may only concern a small percentage of total plasmid DNA, at least for MAR-devoid plasmids. In contrast, our results suggest that a significant proportion of the plasmids might integrate, even without a MAR element. Conceivably, the occurrence of circular plasmids may be underestimated by the damaging of circular plasmids upon extraction, which would result in their degradation by the exonuclease. Although this cannot be formally ruled out, control spiking experiments indicated that degradation of plasmids by the DNase should not exceed 10-20% of the circular molecules. In any case, the integration of electrotransferred DNA upon *in vivo* electrotransfer has now been reported in several independent studies, and this should be taken in consideration when studying potential therapeutic use of DNA electrotransfer for humans.

We find that the total amounts of transgenes persisting 25 days post electrotransfer or later are similar when comparing plasmids that contain or not a MAR element. However, transgene expression persists in the presence of the MAR, while it is gradually silenced in absence of this element. This is unlikely to result from the organization of the regulatory network, as we also observed this effect for constitutive GFP expression in electrotransferred muscles. Previous observations have indicated that the presence of prokaryotic DNA sequences, such as the bacterial antibiotic selection gene, may lead to the formation of heterochromatin and to silencing effects [39]. Consistently, use of plasmid vectors deleted of sequences of bacterial origin, termed minicircles, have been associated with more persistent expression [40]. Our findings illustrate that inclusion of a MAR may also oppose such silencing effects, obviating the need to remove vector sequences. This interpretation is supported by the previous finding that hMAR 1–68 sustains transgene silencing after chromosomal integration, and that it can even act to reverse established silencing effects in cultured cell lines [33]. Here, we observed increasing GFP expression at later times after the electrotransfer of MAR-containing plasmids, suggesting that the MAR elements can promote expression from silent transgenes *in vivo* as well as *in vitro*.

## Conclusions

Taken together, our findings indicate that MAR elements may be used to mediate persistent and more reliable regulated or constitutive expression in differentiated tissues *in vivo*, although this may imply an increased frequency of genomic integration of the transgenes.

## Methods

### Ethics statement

This study was carried out in strict accordance with the Swiss guidelines for the care and cure of animals (i.e. the Federal law on the protection of animals, and the Ordinance on the protection of animals). The protocol was approved by the Animal Experimentation Committee of the Veterinary Service of Canton de Vaud (Permit Number: VD1962). All blood sampling was performed under isoflurane anesthesia, and all efforts were made to minimize suffering.

### Plasmids constructs

All plasmids were generated using standard techniques and verified by sequencing. The pTet-ON™ and pTet-Off™ plasmids were obtained from Clontech. The TetR coding region of pTet-Off™ was fused to the SV40 large T antigen nuclear localisation signal (NLS) and to various transcriptional repression domains, as described previously [9]. The coding sequences for the fusions repressors were inserted in the pCAGGS expression vector under the control of chicken β actin CAG promoter (kindly provided by Jun-ichi Miyazaki, University of Tokio; [41]). Reporter plasmids were previously described [6]. The vector used to express the transcriptional activator Gal4VV was generated from pGAL-VP16 [6] by replacing the full length VP16 activation domain by a dimer of its minimal acidic activation domain [42]. The humanised version of TetR (hTetR) was kindly provided by W. Reith [24]. The final combination of elements of the network vectors were termed CAG-hTetR-TR450W for the repressor, 5XGal4-7XtetO-Gal4VV for the activator, and 5XGal4-7XtetO for the reporter or therapeutic constructs.

Plasmids containing the utrophin, erythropoietin or the luciferase cDNA subcloned under the control of the 5Xgal4-7XtetO inducible promoter of the network system where used where indicated. The human MAR 1–68 was cloned upstream of the inducible promoter in the network activator and reporter constructs where indicated. MAR 1–68, or another human MAR, MAR X-29, were placed upstream of the SV40 promoter driving eGFP constitutive expression, for comparison to the parental MAR-devoid construct.

### DNA purification for electrotransfer

Pellets of overnight 1 liter bacterial cultures were resuspended in Glucose/Tris/EDTA (GET) and freshly prepared 0.2 M NaOH/1% SDS solution was added for alkaline lysis. Neutralization was done with 3 M potassium acetate solution (pH 5.5) and DNA was then precipitated with 0.6 vol isopropanol. The DNA pellet was resuspended in TE buffer, and 1 g of CsCl per ml of solution was added. The solution was transferred to a 12 ml ultracentrifuge tube, to which ethidium bromide was added before sealing 100 μl of 10 mg/ml Tubes were centrifuged in a Beckman ultracentrifuge with a Vti90 rotor at 79000 rpm for 13.5 hours. The supercoiled plasmid DNA band was collected and the ultracentrifugation was repeated. After DNA band collection, ethidium bromide was removed by extraction with an equal volume of NaCl-saturated isopropanol. Plasmid DNA was dialyzed against 150 mM NaCl.

### Intramuscular electrotransfer of genes and doxycycline administration

*In vivo* experiments were carried out on 5–6 week-old C57BL6 female mice (Iffa Credo-Charles River, France) or *mdx*5cv (in-house animal facility). Animals were housed in a facility with food and water *ad libitum*.

The tibialis anterior muscles were pre-treated with hyaluronidase 2–4 h before plasmid injection using a Hamilton syringe. The muscles were injected with 0.8 U/μl of bovine hyaluronidase in 25 μl of normal saline [28]. Plasmid DNA (1 μg/μl) in normal saline was injected intramuscularly. All injections (30 μl) were carried out inside the tibialis anterior muscle, under Ketaminol (100 mg/kg) and Narcoxyl (10 mg/kg) sedation. Following the intramuscular injection of DNA, an electrical field was applied to the muscle. The electrodes were applied to the medial and lateral sides of the lower hind limb and electrode jelly was used on the electrode to ensure good electrical contact. A voltage of 200 V/cm was applied in 8 ms pulses at 1Hz with a BTX electroporator. Doxycycline-HCl (SIGMA) was dissolved in drinking water at final concentration of 400 μg/ml, 3% (wt/vol) sucrose and 4% ethanol, which was provided *ad libitum* to the mice. The proportions of repressor, activator and reporter constructs were 0.05 μg, 15 μg and 15 μg respectively in the final version of the hTetR-TR450W-based network system. No deleterious effect of plasmid injection or of the electrotransfer process was observed, as observed from the occurrence of centrally located nuclei with muscle sections (Additional file 4).

### Luciferase, renilla and EPO assays

After sacrifice of the mice by cervical dislocation, muscles were collected two weeks after the electrotransfer and resuspended in 2 ml of Passive lysis buffer (PROMEGA) with the addition of the Protease Inhibitor Cocktail tablets (Roche) and homogenized by Ultra-thurax (Heidolph). Samples were centrifuged for 10 min at 12,000 rpm, and 10 μl supernatants were tested in duplicates for luciferase and renilla activity using the Dual-Luciferase® Reporter Assay System (PROMEGA).

Blood samples were collected by retro-orbital puncture of mice anesthetized with 5% Isofluorane. The separated plasma was used for murine EPO determination by the Quantikine enzyme linked immunosorbent assay (R&D Systems).

### AFM and histological assays

The processing of tibialis anterior muscles in thick longitudinal sections and AFM assays were as described previously [29]. We used Mann–Whitney test to compare the distributions and they were scored as statistically significantly different with p < 0.05. For GFP quantification, tibialis anterior muscles were transversally sectioned at 10 μm thickness and incubated 1 hour with Alexa Fluor 594-labelled Wheat Germ Agglutinin (2 μg/ml) in PBS (Molecular Probes w-11262), to stain the extracellular matrix glycoproteins. After 3 washes in PBS, slides were mounted with VECTASHIELD® Mounting Medium. The fluorescence of the Green Fluorescent Protein was directly assessed by fluorescence microscopy in the sarcoplasma of muscle fibers and was quantified using the Tissue Quest image analysis software (Tissue Gnostics, Vienna, Austria). Between 2000 and 3000 fibers per slides were analyzed and the mean fluorescence was recorded.

### Purification of total DNA from muscles and quantification of episomal plasmids

Total DNA was purified from murine muscle explants using the Blood & Cell Culture DNA Mini Kit (Qiagen) following the manufacturer’s instructions. Elution of the extracted total DNA was performed in 200 μl of water. Transformation was performed with 1 μl of the total DNA solution added to 25 μl of ultra electro-competent cells DH-10B (Invitrogen). After a 2 kV electrical pulse, bacterial cells were incubated in LB for 1 h at 37°C and plated on ampicilline-containing LB AGAR plates.

### DNA quantitative PCR assays

Qantitative PCR was performed in 15 μl containing 175 ng of total genomic DNA extracted from eletrotransferred mice muscles, or as otherwise indicated in the figure legends. 40 cycles of amplification were performed using GFP (AGCAAAGACCCCAACGAGAA and GGCGGGGGTCACGAA) oligonucleotides as primers, and the mouse GAPDH primers (CGACCCCTTCATTGACCTC and CTCCACGACATACTCAGCACC) as a reference.

### Plasmid Safe DNase treatment and qPCR

2.5 μg of total mouse muscle genomic DNA was digested with 100 U Plasmid safe DNase, 5 μl of ATP (100 mM) in 500 μl for 8 hours at 37°C. This was followed by the addition of an additional 100 U of Plasmid Safe DNase and 5 μl of ATP (100 mM), followed by an over-night incubation. When processing the genomic DNA extracted from plasmid-electrotransferred muscles, 50% of the extract was diluted to 500 μl, and two additional cycles of treatment with 100 U of Plasmid Safe DNase were performed as before, to ensure the total degradation of the chromosomal or damaged plasmid DNA. 7 μl of the digestion mix (containg 30 ng of total DNA) was processed to quantify the GFP and GAPDH undigested DNAs in 15 μl qPCR reactions. The copy number of the GFP transgene was normalized to the copy number of the GAPDH chromosomal gene prior to treatment with the DNase, taking in consideration the average efficiency values of the GFP and GAPDH amplification reactions as described previously [43]. Because we observed that inclusion of the PS-DNAse buffer lowered the qPCR-mendiated quantification of the GAPDH reference gene, a correction factor of 0.1 was applied to data from qPCR reactions that contained the PS-DNase mix, so as to reflect the true DNA quantities as determined in control titration reactions with or without the PS-DNase mix (Figure 5B).

## Competing interests

SP, RWvZ, DS, ML, FH and AJK declare no competing interest. NM owns share and acts as a consultant of a company, Selexis SA, that uses proprietary DNA elements to mediate protein expression *in vitro.*

## Authors’ contributions

SP, RWvZ, DS, ML and FH performed and interpreted the experiments. SP, RWvZ and NM wrote the manscript. ML, AJK and NM contributed to the study design and coordination, and to the interpretation of results*.* All authors approved the manuscript.

## Supplementary Material

Additional file 1 **Dose–response titration of the EF-1α-hTETR-TR450W repressor vector.** Constant amounts (10 μg) of the activator (5XGTTIGal-VV) and reporter gene (5XGTTI-luc) expression plasmids were introduced into mouse tibialis anterior muscles together with a renilla reference plasmid and varying amounts of the repressor protein expression vector (EF-1a-hTETR-TR450W), as indicated. Analysis of the expression levels was performed 10 days after the electrotransfer, from muscle extracts obtained from mice provided of not with doxycycline in the drinking water. Click here for file

Additional file 2 **Plasmid transformation efficiency.** The transformation efficiency of GFP expressing vectors containing or not the indicated MAR element, as used in Figures 3 and 4C, was compared to that of the smaller parental pUC plasmid. 10 ng of plasmids were mixed with 100 ng of total genomic DNA extracted from muscles not subjected to an electrotransfer, and the mixes were added to electrocompetent bacterial cells for transformation by episomal plasmids as described in the Methods. The star sign indicates statistical significance (p < 0.05) while ns stands for non-significant. Click here for file

Additional file 3 **Real-time PCR quantification of genome-integrated, linear or circular episomal GFP DNA.** Total genomic DNA was extracted from either non-injected mouse tibialis anterior muscles, or from primary murine mesioangioblast muscle-precursor cells having stably integrated approximately 4 copies of the GFP coding sequence into one of their chromosomes. 10 ng of genomic DNA were mixed or not with 4 pg of supercoiled circular or linearized GFP expression plasmid, as indicated, which corresponds to approximately 430 copies of the 4.3 kb GFP-coding plasmid per diploid genome. The mix was incubated twice with 100 units of the Plasmid Safe (PS) DNAse during 12 h, in two consecutive incubations, to assess the plasmid resistance to degradation. Alternatively, the genomic DNA was similarly incubated twice for 12 h with the PS-DNAse, and the circular plasmid was added at the end of the incubations just before DNA purification and quantification of the GFP sequence by qPCR. The GFP gene copy number was normalized relative to that of the genomic GAPDH gene (prior to digestion), and it is represented as the GFP sequence copy number per diploid genome, assuming a total number of 660 GAPDH gene and pseudogene copies per diploid genome (Liu et al., 2009). Values lower than 0.1 GFP copies per genome indicate background noise values, as obtained with the genomic DNA devoid of GFP sequence as in the first two lanes. Over 90% of the supercoiled plasmids incubated with the DNAse remained undigested, whereas linearized plasmids or chromosomally-integrated GFP transgenes are digested to near near completion (99%) by the PS-DNAse. Click here for file

Additional file 4 **Quantification of central nuclei in WT and *mdx *muscle fibers following electrotransfer.** We quantified the number of central nuclei (representing regenerating muscle) in WT and *mdx* mice, electrotransferred or not with DNA, 7 days after the experiment. Cryostat muscle sections were stained by hematoxyline/eosine method, and the location of nuclei was quantified by microscopy. No significant effect of electroporation or of the DNA was detected from 3 different muscle sections for each condition. Click here for file
