# Supplementary material for: MAR-mediated integration of plasmid vectors for in vivo gene transfer and regulation
Source: BMC Mol Biol. 2013 Dec 2;14:26. doi: 10.1186/1471-2199-14-26 (PMC4219123; doi:10.1186/1471-2199-14-26)
Supplement: Additional file 1 — Dose–response titration of the EF-1α-hTETR-TR450W repressor vector. Constant amounts (10 μg) of the activator (5XGTTIGal-VV) and reporter gene (5XGTTI-luc) expression plasmids were introduced into mouse tibialis anterior muscles together with a renilla reference plasmid and varying amounts of the repressor protein expression vector (EF-1a-hTETR-TR450W), as indicated. Analysis of the expression levels was performed 10 days after the electrotransfer, from muscle extracts obtained from mice provided of not with doxycycline in the drinking water. [file 1471-2199-14-26-S1.pdf]

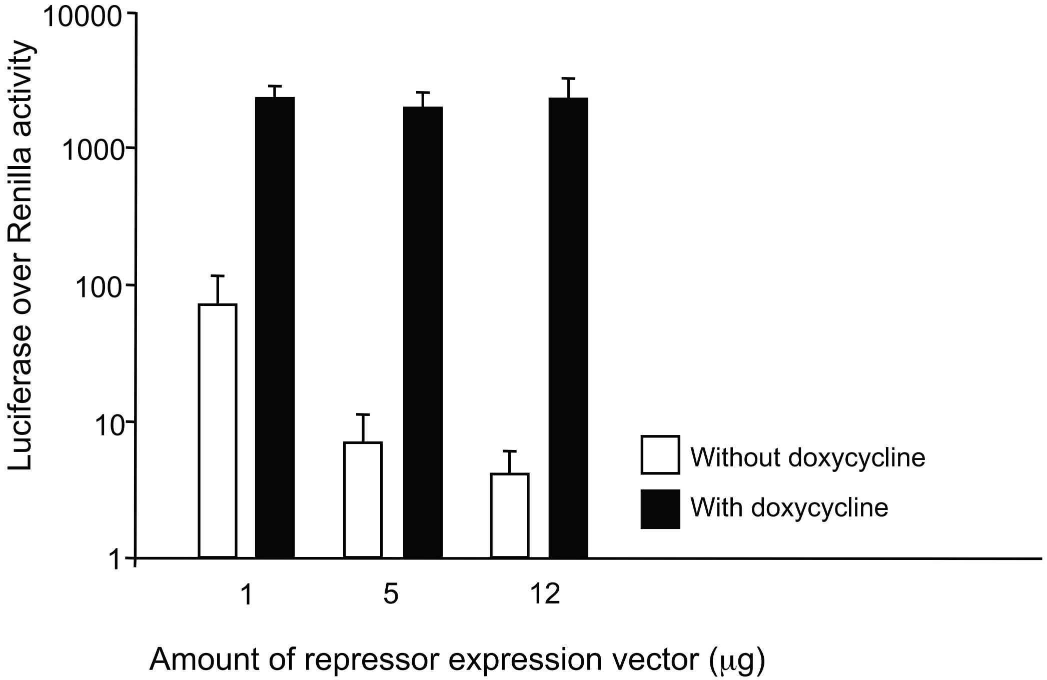

Additional file 1. Dose-response titration of the EF-1α-hTETR-TR450W repressor vector. Constant amounts (10 μg) of the activator (5XGTTIGal-VV) and reporter gene (5XGTTI-luc) expression plasmids were introduced into mouse tibialis anterior muscles together with a renilla reference plasmid and varying amounts of the repressor protein expression vector (EF-1a-hTETR-TR450W), as indicated. Analysis of the expression levels was performed 10 days after the electrotransfer, from muscle extracts obtained from mice provided of not with doxycycline in the drinking water. Luciferase or renilla activity were undetectable from non-electrotransferred negative control muscles in these assay conditions.

Puttini et al.
