# Supplementary material for: MAR-mediated integration of plasmid vectors for in vivo gene transfer and regulation
Source: BMC Mol Biol. 2013 Dec 2;14:26. doi: 10.1186/1471-2199-14-26 (PMC4219123; doi:10.1186/1471-2199-14-26)
Supplement: Additional file 2 — Plasmid transformation efficiency. The transformation efficiency of GFP expressing vectors containing or not the indicated MAR element, as used in Figures 3 and 4C, was compared to that of the smaller parental pUC plasmid. 10 ng of plasmids were mixed with 100 ng of total genomic DNA extracted from muscles not subjected to an electrotransfer, and the mixes were added to electrocompetent bacterial cells for transformation by episomal plasmids as described in the Methods. The star sign indicates statistical significance (p < 0.05) while ns stands for non-significant. [file 1471-2199-14-26-S2.pdf]

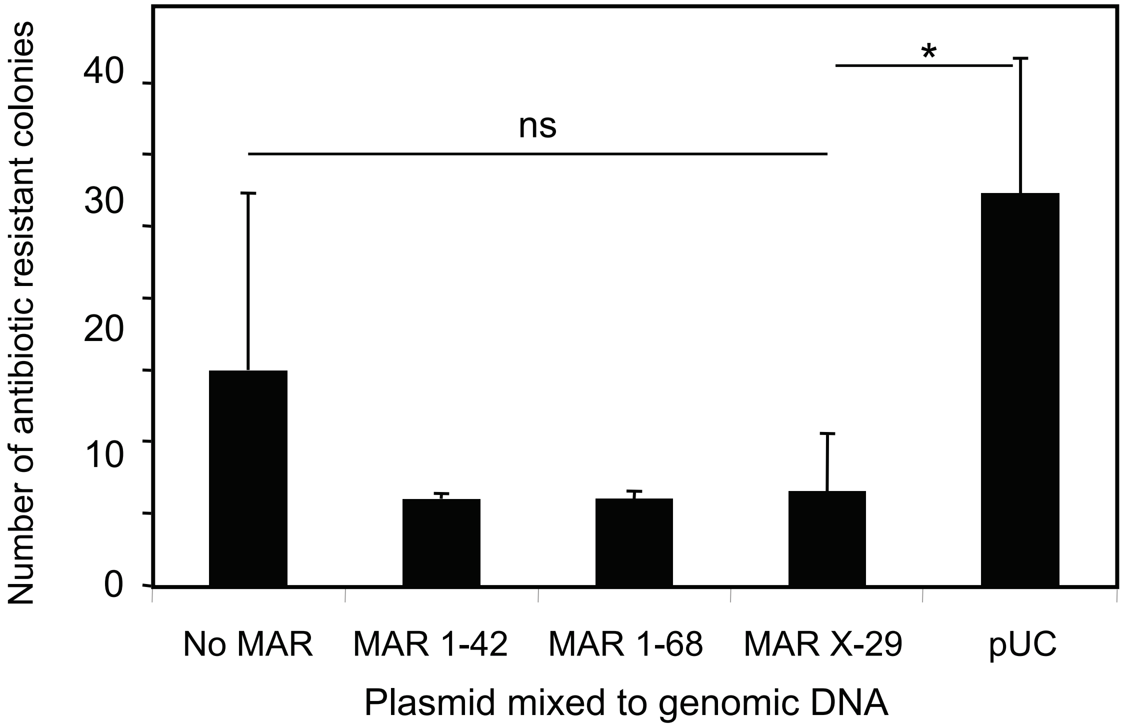

Additional file 2. Assay of plasmid transformation efficiency.
The transformation efficiency of GFP expressing vectors containing or not the indicated MAR element, as used in Figure 3 and 4C, was compared to that of the smaller parental pUC plasmid. 10 ng of plasmids were mixed with 100 ng of total genomic DNA extracted from muscles not subjected to an electrotransfer, and the mixes were added to electrocompetent bacterial cells for transformation by episomal plasmids as described in the Materials and Methods. The star sign indicates statistical significance ($p<0.05$) while ns stands for non-significant.

Puttini et al.
