# Supplementary material for: MAR-mediated integration of plasmid vectors for in vivo gene transfer and regulation
Source: BMC Mol Biol. 2013 Dec 2;14:26. doi: 10.1186/1471-2199-14-26 (PMC4219123; doi:10.1186/1471-2199-14-26)
Supplement: Additional file 3 — Real-time PCR quantification of genome-integrated, linear or circular episomal GFP DNA. Total genomic DNA was extracted from either non-injected mouse tibialis anterior muscles, or from primary murine mesioangioblast muscle-precursor cells having stably integrated approximately 4 copies of the GFP coding sequence into one of their chromosomes. 10 ng of genomic DNA were mixed or not with 4 pg of supercoiled circular or linearized GFP expression plasmid, as indicated, which corresponds to approximately 430 copies of the 4.3 kb GFP-coding plasmid per diploid genome. The mix was incubated twice with 100 units of the Plasmid Safe (PS) DNAse during 12 h, in two consecutive incubations, to assess the plasmid resistance to degradation. Alternatively, the genomic DNA was similarly incubated twice for 12 h with the PS-DNAse, and the circular plasmid was added at the end of the incubations just before DNA purification and quantification of the GFP sequence by qPCR. The GFP gene copy number was normalized relative to that of the genomic GAPDH gene (prior to digestion), and it is represented as the GFP sequence copy number per diploid genome, assuming a total number of 660 GAPDH gene and pseudogene copies per diploid genome (Liu et al., 2009). Values lower than 0.1 GFP copies per genome indicate background noise values, as obtained with the genomic DNA devoid of GFP sequence as in the first two lanes. Over 90% of the supercoiled plasmids incubated with the DNAse remained undigested, whereas linearized plasmids or chromosomally-integrated GFP transgenes are digested to near near completion (99%) by the PS-DNAse. [file 1471-2199-14-26-S3.pdf]

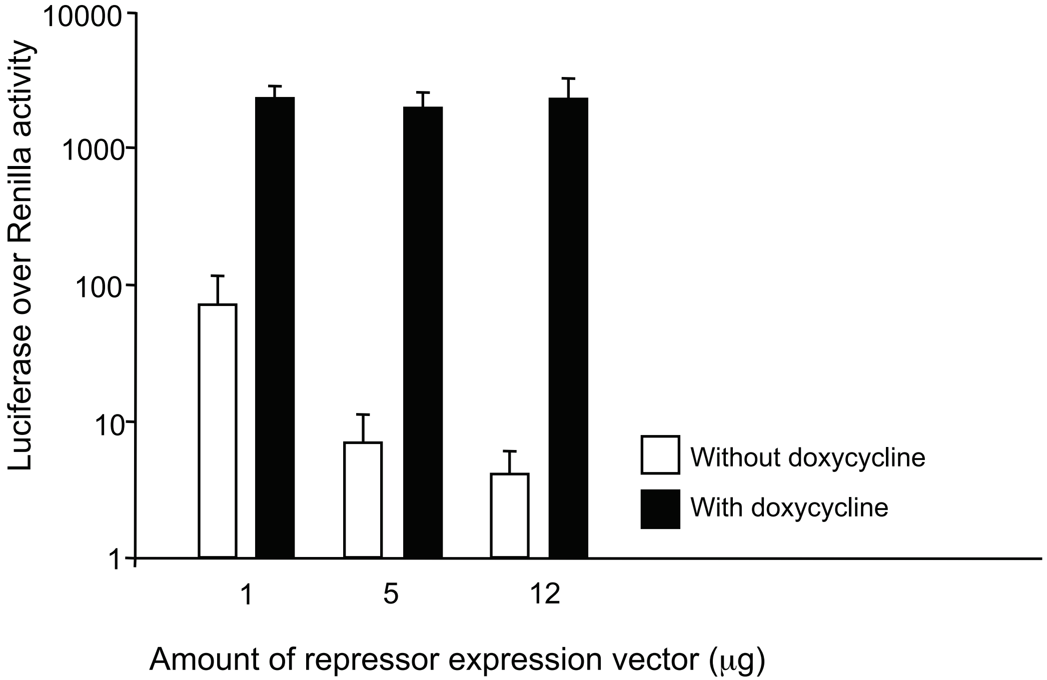

Additional file 1. Dose-response titration of the EF-1α-hTETR-TR450W repressor vector. Constant amounts (10 µg) of the activator (5XGTTIGal-VV) and reporter gene (5XGTTI-luc) expression plasmids were introduced into mouse tibialis anterior muscles together with a renilla reference plasmid and varying amounts of the repressor protein expression vector (EF-1a-hTETR-TR450W), as indicated. Analysis of the expression levels was performed 10 days after the electrotransfer, from muscle extracts obtained from mice provided of not with doxycycline in the drinking water. Luciferase or renilla activity were undetectable from non-electrotransferred negative control muscles in these assay conditions.

Puttini et al.

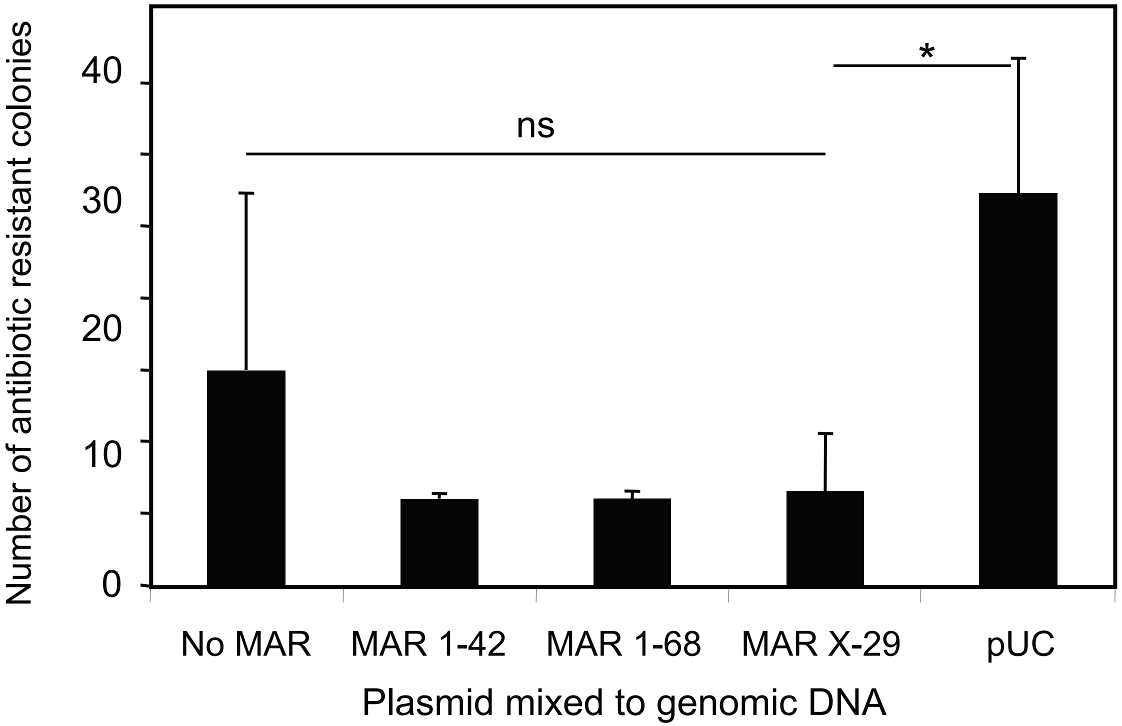

Additional file 2. Assay of plasmid transformation efficiency.
The transformation efficiency of GFP expressing vectors containing or not the indicated MAR element, as used in Figure 3 and 4C, was compared to that of the smaller parental pUC plasmid. 10 ng of plasmids were mixed with 100 ng of total genomic DNA extracted from muscles not subjected to an electrotransfer, and the mixes were added to electrocompetent bacterial cells for transformation by episomal plasmids as described in the Materials and Methods. The star sign indicates statistical significance ($p<0.05$) while ns stands for non-significant.

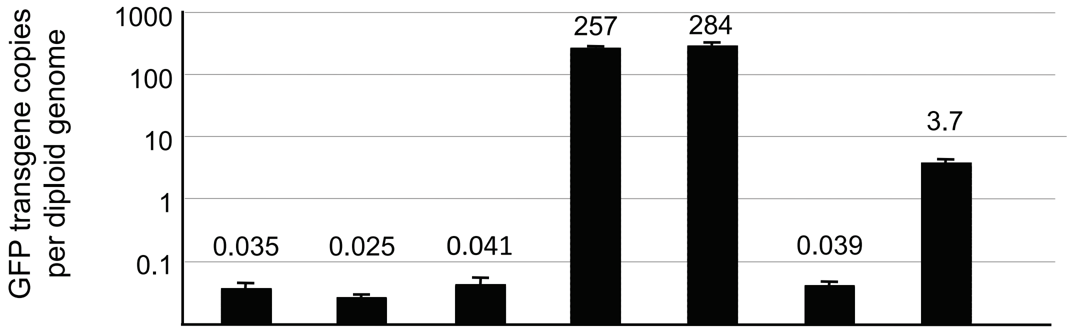

Genomic DNA (ng)	10	10	10	10	10	-	-
GFP sequence-integrated genomic DNA (ng)	-	-	-	-	-	10	10
Linear plasmid (pg)	-	-	4	-	-	-	-
Circular plasmid (pg)	-	-	-	4	-	-	-
Plasmid safe DNAse during incubations (U)	-	2x100	2x100	2x100	2x100	2x100	-
Circular plasmid (pg, added after the incubations)	-	-	-	-	4	-	-

Additional file 3. Real-time PCR quantification of genome-integrated, linear or circular episomal GFP gene DNA.
Total genomic DNA was extracted from either non-injected mouse tibialis anterior muscles, or from primary murine mesioangioblast muscle-precursor cells having stably integrated approximately 4 copies of the GFP coding sequence into one of their chromosomes. 10 ng of genomic DNA were mixed or not with 4 pg of supercoiled circular or linearized GFP expression plasmid, as indicated, which corresponds to approximately 430 copies of the 4.3 kb GFP-coding plasmid per diploid genome. The mix was incubated twice with 100 units of the Plasmid Safe (PS) DNAse during 12h, in two consecutive incubations, to assess the plasmid resistance to degradation. Alternatively, the genomic DNA was similarly incubated twice for 12h with the PS-DNAse, and the circular plasmid was added at the end of the incubations just before DNA purification and quantification of the GFP sequence by qPCR. The GFP gene copy number was normalized relative to that of the genomic GAPDH gene (prior to digestion), and it is represented as the GFP sequence copy number per diploid genome, assuming a total number of 660 GAPDH gene and pseudogene copies per diploid genome (Liu et al., 2009). Values lower than 0.1 GFP copies per genome indicate background noise values, as obtained with the genomic DNA devoid of GFP sequence as in the first two lanes. Over 90% of the supercoiled plasmids incubated with the DNAse remained undigested, whereas linearized plasmids or chromosomally-integrated GFP transgenes are digested to near near completion (99%) by the PS-DNAse.

Puttini et al.

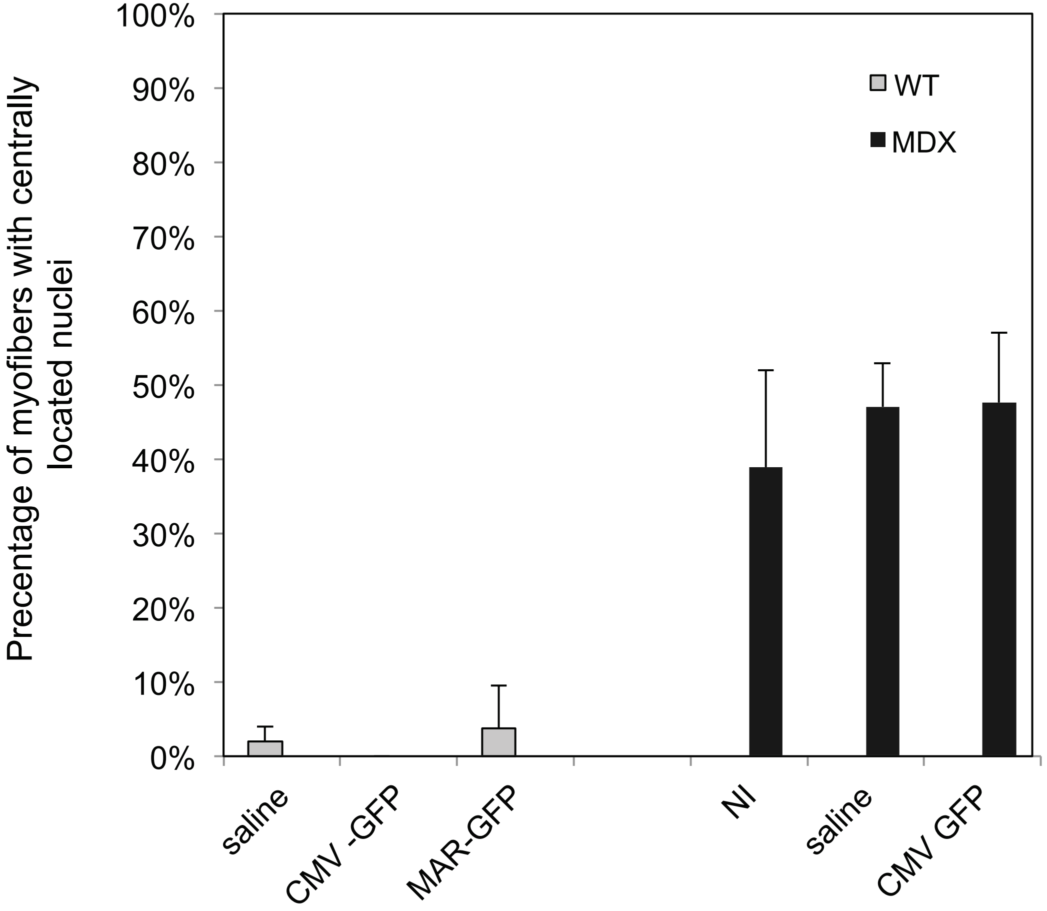

Additional file 4. Quantification of central nuclei in WT and *mdx* muscle fibers following electrotransfer. We quantified the number of central nuclei (representing regenerating muscle) in WT and *mdx* mice, electrotransferred or not with DNA, 7 days after the experiment. Cryostat muscle sections were stained by hematoxyline/eosine method, and the location of nuclei was quantified by microscopy. No significant effect of electroporation or of the DNA was detected from 3 different muscle sections for each condition.
